# Social vulnerability assessment of dog intake location data as a planning tool for community health program development: A case study in Athens-Clarke County, GA, 2014-2016

**DOI:** 10.1371/journal.pone.0225282

**Published:** 2019-12-02

**Authors:** Jessie L. Dyer, Lisa Milot

**Affiliations:** 1 Department of Small Animal Medicine and Surgery, College of Veterinary Medicine, University of Georgia, Athens, Georgia, United States of America; 2 Athenspets, Inc., Athens, Georgia, United States of America; 3 School of Law, University of Georgia, Athens, Georgia, United States of America; Ehime University Graduate School of Medicine, JAPAN

## Abstract

A retrospective spatial analysis of dog intake data from an open admission animal shelter in Georgia was conducted to explore patterns within dog demographics and outcomes by pickup location or by the home address of the person who transferred ownership rights of the dog to Athens-Clarke County Animal Control during the period 2014–2016. Spatial analysis found the relationship between these intake locations and the final disposition of the dogs to be non-random, suggesting social and environmental influences on distribution. Statistically significant clusters were identified using the Getis-Ord Gi* statistic. This study found statistically significant hot spots (i.e., areas with higher than expected values) and cold spots (i.e., areas with lower than expected values) for the intake of dogs with known health issues, physically neglected dogs, juveniles, and adults. Only statistically significant hot spots were found for socially neglected dogs and dogs whose final disposition was euthanasia due to severe health or behavioral issues. Given the close relationship between humans and dogs, this study explores the association of impounded dog clusters and a previously developed social vulnerability index. Social vulnerability is the product of social inequalities and inequalities related to the human-built environment. The social vulnerability index provides one tool for understanding the differences in characteristics of dogs from different intake locations. Results of this study indicate the utility of non-animal focused data as a planning tool for community programs and to allow for efficient allocation of limited resources for veterinary and other community outreach programs.

## Introduction

Despite long-term interventions and significant increase in live release rates, the mass euthanasia of dogs in animal shelters in the United States has continued to be a problem [1]. An estimated 800,000 dogs are euthanized annually in U.S. shelters and state legislatures have recognized it as a severe crisis [2]. Companion animals in excess of human needs are often euthanized due to population pressures (i.e., lack of resources to adequately provide care). Many animal shelters lack the resources needed to adequately provide care for all of the companion animals produced in their communities that do not have stable homes [3]. Sexual altering, or neutering, has been shown to successfully reduce companion animal populations over an extended period of time within targeted locations [4, 5].

However, a community’s production and abandonment of excess companion animals is not the only reason shelters euthanize animals. Many animals arrive at animal shelters with health issues associated with human neglect or with severe behavioral issues [5, 6]. Even with companion animal population reduction through large-scale sexual altering, euthanasia of animals with health issues associated with human neglect or severe behavioral issues from social neglect will continue if successful interventions do not occur before intake [7].

One challenge to such interventions is the lack of collection and standardization of animal shelter data. Although great strides have been made towards the collection and standardization of animal shelter data, databases comparable to the U.S. Census do not exist. Animal shelter data was called a “statistical black hole” as recently as the 1990s [8]. A renewed effort to understand pet population statistics led to the formation of the National Council on Pet Population Study and Policy (NCPPSP) in 1994 [9]. Discussions by NCPPSP led to the collaboration between national animal welfare organizations to develop Shelter Animals Count, a national voluntary shelter animal intake and disposition database in 2016. We anticipate that the Shelter Animals Count data set will be invaluable to researchers in the coming years. Until then, animal shelter data can be expected to remain unavailable or inconsistent.

When categorized by approach to admitting animals, there are two primary types of animal shelters within the U.S. “Open admission” shelters are institutions that accept all animals within a subject class (e.g., domesticated animals and injured wild animals) from the areas they serve, regardless of the animal’s physical or mental condition or the availability of housing space. In addition, they generally take in stray animals: those presumed to have an owner, but where the owner is either unknown, nonexistent, or does not surrender his rights upon intake. For these animals, the shelter performs a title-clearing function; animals not reclaimed by an owner during a statutory stray hold period are deemed abandoned and become the property of the shelter which can then transfer title to a new owner without the consent of the prior owner (if any). Open admission shelters may euthanize animals that are neither behaviorally dangerous nor critically ill when the impounded animal population exceeds the available space and resources.

In contrast, “limited admission” shelters, sometimes referred to as “no-kill,” can turn away animals deemed “non-adoptable” (or less adoptable) by the organization [10]. The non-adoptable or less adoptable category may include those animals that have estimated high anticipated veterinary costs, are of advanced age, present with even minor behavioral issues, or have the physical traits of stigmatized breeds. Animal admission into a limited admission shelter is often a business decision, as animals that appear more adoptable at intake generally have a higher turnover, incur lower costs, and result in greater revenue than those that initially appear less adoptable or non-adoptable. Moreover, a limited admission shelter may be legally prohibited from taking in stray animals. These animals often enter open admission shelters. Thus, by admitting all animals within covered species located within the geographic territory, the population at an open admission shelter reflects the range of dogs that are homeless in each community in a way that the population at limited admission shelters does not.

Some shelters use a blended admission model in which stray animals are admitted on an open admission basis, while owner-surrendered animals are only selectively admitted. For example, DeKalb County Animal Services (DeKalb County, Georgia) is a publicly funded shelter that impounds stray animals within county boundaries which has contracted Lifeline Animal Project to manage its shelter. However, it “generally only accept[s] adoptable pets so if your pet is elderly, has behavior issues or is sick it is your responsibility to take it to a veterinarian for treatment, hire a trainer or have it euthanized at a private veterinarian. During times of high intake or when our shelter is full, accepting owner-surrendered animals may be completely suspended” [11].

Upon intake of dogs at an open admission shelter, variance within the physical and social conditions of dogs reflects differing welfare standards, including the provision of food and housing, and social norms, such as the availability and awareness of veterinary practices and basic pet care. Animal welfare standards include how and for how long dogs are confined in their prior home and whether they are housed inside a house or outside with adequate cover; if they are microchipped and/or regularly wear a collar with identification; whether they are sexually intact or altered; whether humans in the household engage with them in obedience training and ensure that their exercise needs are met; human knowledge about and provision of adequate nutrition and veterinary care; and the degree and types of socialization between the dogs and humans and other animals.

Dogs impounded at open admission shelters often exhibit poor physical condition or under socialization. Symptoms of poor physical condition include the presence of preventable or treatable conditions such as emaciated body condition, ingrown collars, or the presence of parasites. These conditions may be the result of short term care deficits (e.g., flea infestations and intestinal parasites which become evident almost immediately after infection) or longer-term care deficits (e.g., dangerously low body weight, heartworm infections, collar embedding, and severe skin infections take several weeks to months to manifest).

Social neglect is apparent in open admission dog populations when a dog presents with undesirable responses to unfamiliar situations, such as upon impound and housing at a municipal shelter or at the time of rehoming and may result in injuries to humans or other dogs and/or euthanasia of the neglected dog. Underlying these issues is a poverty of social experiences prior to impound. For example, dogs may react inappropriately if they have spent only limited time with other dogs previously as the subtle signaling of non-verbal communications that is the basis for canine communication may go unnoticed. Research has indicated that tethered dogs are more likely to bite both humans and other dogs than untethered dogs, and dogs that have been segregated from humans through isolated housing methods may display fear or confusion during interactions as social cues are unreciprocated [12, 13]. These behaviors reflect social neglect and may go unreported until dogs become unmanageable or aggressive, at which point they may play a role in the decision by an owner to surrender ownership of a dog to a shelter or to not reclaim a dog picked up as a stray (See Table 1 for more information about these categories).

**Table 1:** Health and behavioral categories of Athens-Clarke County Animal Control dog intake by basic health and behavioral assessment descriptive terms collected upon intake.

Health or behavioral category^a^	Basic health and behavioral assessment term(s)
**Healthy**	healthy; no comment
**Known health issue upon intake**	abscess; allergy; bleeding; blood; bloody; burn; burned; burnt; cherry eye; cough; dead on arrival; diarrhea; died; discharge; hernia; hip problems; hot spot; infection; parasite; rash; tumor;
**Visible health issues typically associated with physical neglect**	broken bones or teeth without additional signs of trauma; collar indentation in flesh; Demodex mange; ear infection; fleas and/or tick infestation; emaciated; gunshot wound; in-grown collar; large tumor; long nails; mange; matted; neglect; obese; overweight; parvo; puncture wound; significant hair loss; skinny; thin; tight collar; underweight; visible skeletal features; yeast infection on skin
**Behaviors typically associated with social neglect**	aggressive; aggression; behavioral assessment outcome; bites; fear biter; reactive; “temperament” description; withdrawn
**Visible health issues typically associated with vehicular trauma**	abrasion; hit by car; road rash; shock; trauma

^a^Dogs may be categorized in more than one category.

Due to the close relationship between humans and dogs in the U.S., the two populations often share physical and social conditions in life. Physical or social neglect in pets may merely be an extension of the same in the human members of a household. Despite this connection, human and animal health fields remain silos due to professional segregation, separation of government regulatory efforts, and funding priorities [14, 15]. Historically, there has been a strong nature/culture divide in western cultures that limits research on environmental factors to the human perspectives and implies that humans are “more” than non-human animals, otherwise known as human exceptionalism [16]. Yet approaches to human-companion animal legal and cultural relationships in U.S. society have been shifting in recent decades from a property paradigm, in which an animal’s worth is based on its functional role or market value, to a guardianship model that recognizes that dogs are living beings with sentimental and other non-economic value to humans [17]. As companion animals increase in societal importance, further consideration of the human role in the social development of dogs and the sizable number of animals that enter open admission shelters within the U.S. is needed [18]. This shift can be seen in the legal transitioning of animals from "property" to quasi-legal actor, at least in some realms. For example, unlike any other form of property, companion animals can be the beneficiaries of trusts in every U.S. state [19].

Given the connection between animal physical and social neglect, human social inequalities, and inequalities within the shared built environment, understanding the link between human vulnerability and dog health may assist when designing veterinary or other companion animal community programs. Spatial cluster analysis of dog intake and disposition data (e.g., dog age, health, and behavior demographics, and outcomes by the location where each dog was found) can identify areas where behavioral interventions and veterinary services are lacking in a community [20–22]. For example, hot spots (i.e., greater than expected values) for juvenile impounds indicate areas with high dog population turnover, low neuter rates, the presence of intentional breeding programs, or high interaction rates between unaltered sexually mature dogs. Information concerning the location of clusters of such dogs is essential information for designing effective interventions; education about the benefits of altering dogs, access to free or low-cost veterinary services, and the provision of materials and labor to house intact animals appropriately can be targeted to these areas.

Moreover, clusters can provide information about the social environments producing the most vulnerable dogs and those at highest risk for euthanasia in the shelter setting. Marginalized and at risk dog populations may be linked to human vulnerability given the relationship between the two populations. Thus, this study examines each dog intake cluster in relation to the social vulnerability index (SVI) of its associated community. The United National Development Programme (UNDP) defines vulnerability as “a human condition or process resulting from physical, social, economic and environmental factors, which determine the likelihood and scale of damage from the impact of a given hazard” [23].

Social vulnerability is the product of social inequalities and inequalities related to the built environment and socially vulnerable populations are less likely to flourish or recover following events (e.g., illness, family restructuring, natural disasters) [24]. Social vulnerability indices have been used within diverse contexts including chronic disease [25], drowning deaths [24], infectious disease [26], mental health [27, 28], nutritional status [29], and physical fitness in children [30]. This study argues that the domains used to measure human social vulnerability apply to dog conditions, as individual characteristics of impounded dogs reflect environmental and social conditions at the community level given the degree to which dogs rely on humans in the U.S.

Given the integration of animals, humans, and the environment, we predicted that the areas with the greatest clusters of (1) impounded dogs, and (2) dogs showing negative health and behavioral variations upon impound would spatially overlap with areas of greatest human social vulnerability. We performed a retrospective spatial analysis of dog intake data collected during 2014–2016 by staff of Athens-Clarke County Animal Control (ACCAC), an open admission animal shelter in Georgia, and explored the utility of using a social vulnerability index (SVI) to explain variations in the geographic distribution of the intake locations of clusters of dogs most at risk in the shelter setting. The results of this study may be used to develop targeted veterinary community outreach programs (such as neutering or vaccination campaigns), human behavioral interventions (such as basic pet care education), and programs that provide the resources needed to better ensure that dogs’ physical and social needs can be met (such as appropriate kenneling and fencing so that dogs become removed from tethers). These programs may reduce the number of dogs that lose their homes while improving the outcomes of the dogs that do enter the shelter setting.

## Materials and methods

### Study area

This study was conducted in Athens-Clarke County, the smallest county in Georgia and home to its fifth most populated city, Athens [31]. Athens-Clarke County is a predominantly urban area that had a population of 123,912 permanent residents in 2015 and has a large transient student population associated with the University of Georgia [31]. Within Athens-Clarke County, 37.8% of the population lived below the poverty level in 2010 [31], compared to a national average of 15.1% [32]. Athens-Clarke County Animal Control (ACCAC) “protects the public safety and health from at-large and dangerous animals and protects animals from inhumane treatment” [33]. As an open admission shelter, ACCAC accepts all dogs, among other animals, brought to the shelter by residents of Athens-Clarke County or those retrieved within county boundaries by ACCAC staff. One of ACCAC’s duties is collecting information about and maintaining a database on the animals housed at its facility. Dog intake data collected by ACCAC staff during 2014–2016 was used in this study.

### Data

For this study, data was requested from ACCAC on dog intake cases from January 1, 2014–December 31, 2016. This study excluded dogs for which the intake location was not available and those with intake locations outside of Athens-Clarke County. Dogs with intake locations outside of Athens-Clarke County were picked up by an Athens-Clarke County resident outside of county boundaries and brought to ACCAC. When a dog arrives at ACCAC, he or she is categorized by intake method (i.e., dog retrieved in the field that was not surrendered; dog surrendered by its owner at the ACCAC facility or in the field; dog brought to the ACCAC facility by a member of the public that is not surrendered; or dog that was trapped). Animals are weighed and assigned a unique identifying number. Age is estimated based on dentition pattern eruption and appearance (i.e., enamel wear patterns and coloration). Staff performs basic health assessments on all dogs at intake through a visual inspection. Visible injuries, the presence of ectoparasites, and other negative health conditions are noted. As of 2016, owner surrendered dogs are tested for heartworms by an antigenic snap test during impound. Many of the stray dogs are tested for heartworms by an antigenic snap test upon the expiration of the stray hold period. The stray hold period is a span of time after which an owner may no longer claim ownership (i.e., reclaim the dog). Stray hold periods are county mandated and vary considerably by location; in Athens-Clarke County, the stray hold period is 5 business days. Health categories for this study were developed from these basic health assessments and resulted in four categories: healthy, known health issues upon intake, visible health issues typically associated with physical neglect, and visible health issues typically associated with vehicular trauma (Table 1).

Indications of behavioral responses to observed stimuli are recorded during impound. These stimuli include reaction to animal control professionals upon initial introduction in the field or at the office; animal reaction to restraint during impound for vaccination, testing, and deworming; reactions in later interactions with staff and shelter volunteers while the dog is kenneled or being handled; and, as warranted by other observations, direct interaction with a range of other dogs. Dogs exhibiting poor socialization behaviors, such as overreaction to humans or dogs beyond that expected within the normal behavior spectrum (reactivity) are categorized as socially neglected for purposes of this study (Table 1).

Other variables of interest for this study are each dog’s age and weight, date of intake, intake location, impound method, behavioral and health assessment, and final disposition. For this study, dogs were categorized as juveniles, adults, or seniors based on age estimates at the time of intake. Juveniles are those dogs that were estimated to be younger than one year of age at intake, adults were estimated to be one year of age or older but no more than six years of age, and seniors were estimated to be greater than six years of age. Each dog’s weight at the time of impound determined size group. Extra small dogs are those that weighed up to 11.9 lbs. at intake, small dogs were 12.0 to 24.9 lbs., medium dogs were 25.0 to 49.9 lbs., large dogs were 50.0 to 99.9 lbs., and extra-large dogs were those that weighed 100 lbs. or more. Dispositions were categorized as adoption, reclaim of stray dogs by owner, transfers to animal rescue groups, euthanasia in the shelter setting or at a veterinarian’s office after intake, and, in rare occurrences, “other.” The category of “other” includes instances in which a dog died during intake or while at the shelter (e.g., from illness) and dogs that escaped or were stolen from the shelter.

### Social vulnerability index

This study utilizes the publicly available SVI developed by the Centers for Disease Control and Prevention, National Center for Environmental Health, Office of Terrorism Preparedness and Emergency Response in collaboration with the Agency for Toxic Substances and Disease Registry’s Geospatial Research, Analysis, and Services Program as a component of a disaster management risk equation [24]. The SVI utilizes U.S. Census data to determine social vulnerability percentile rank within the state of Georgia by census tract [24]. Census-tract populations remain relatively stable across counties [24]. Measures of social vulnerability highlight environmental and social conditions at the community level [24]. A higher percentile rank represents greater social vulnerability.

The domains used to develop the SVI encompass the shared physical and social environment expected of multispecies interactions, such as the human-dog relationship. The four domains used to form the basis of the SVI are Socioeconomic Status (i.e., income, poverty, employment, and education variables), Household Composition/Disability (i.e., age structure, single parenting, and disability), Minority Status/Language (i.e., self-identified race, ethnicity, and English-language proficiency), and Housing/Transportation (i.e., housing structure, population density, and transportation access). The overall SVI is the assessment of all four domain ranks. Many of the domain indicators affect all members of a household, including non-humans.

Socioeconomic status is an important indicator of multispecies vulnerability because it reflects available household assets. Information on socioeconomic status captured within the census reflects the monetary value of the owned property and may represent a larger proportion of total household assets [24]. Income and other resource limitations may inhibit a household’s ability to provide basic needs of companion animals (i.e., dogs, cats, rabbits, etc.) and lead to items being deemed non-vital or allocated a low priority. Socioeconomic status should not be equated with better care of companion animals as higher socioeconomic status may indicate greater available resources without indications additional allocations for companion animals. For this study, associations between the number of owned dogs and the socioeconomic status of owners were not explored.

Household compositions are relevant to companion animal health because of generational views on the household status of dogs, ideas of suitable human-dog and dog-dog interactions, and expectations of a dog’s utility. The household composition includes variables for “dependent children less than 18 years of age, persons aged 65 years and older, individuals with disabilities, and single-parent households” [24]. A study on the empathic response to animal suffering found greater generational similarities in such responses than between multiple generations within the same family, indicating generational views on an animal’s place in society [34].

Minority status and language were included as an SVI domain as they represent social marginalization of racial and ethnic groups; this marginalization may render these populations more vulnerable [24]. For this study, minority status and language may represent communication barriers that may hinder community outreach initiatives and may indicate cultural perceptions of animal welfare and community needs. A recent survey of Latino pet owners found that a large proportion of respondents describing themselves as speaking English “well” or “very well” preferred to receive pet health information in their native language (Spanish) [35]. Bilingual pet health information is not always available or offered by community outreach programs.

Owners provide housing, nutrition, and transportation for their dogs, making the fulfillment of dogs’ needs reliant on human capacity and circumstances. Housing construction and density are associated with personal wealth, with wealthier households spaced farther apart, and the density and quality of housing construction often clusters [24]. Since dog population estimates are calculated from human population estimates, dog population estimates increase as human populations increase [36]. Crowding of both species may lead to attempts to enforce isolation from members of the same species, with a greater likelihood of social conflicts and opportunities for disease transmission when interactions do occur. There may be geographic isolation from necessary services in areas restricted to residential development, limiting access to veterinary offices and pet supply stores to households that own or have easy access to motor vehicles. Even where a vehicle is available, the expense and potential legal issues associated with owning or driving motor vehicles, including insurance, registration, and driver’s license requirements, may lead more vulnerable populations to rely public modes of transportation which prohibit dog transportation. This limitation exacerbates financial issues surrounding access to veterinary care and other necessary pet services and supplies for these populations.

### Spatial analysis

Reported dog intake locations were geocoded using ArcMap (ESRI, ArcGIS, Version 10.4.1). GPS coordinates of dog intakes were projected onto 2010 Topologically Integrated Geographic Encoding and Referencing (TIGER) U.S. shapefiles [36]. The Integrate and Collect Events method aggregated the geocoded locations of dog intake cases to account for point projection variance.

To determine statistically significant hot spot and cold spot spatial clusters, the Getis-Ord Gi* statistic was calculated for each defined characteristic and resultant z-scores, p-values, and confidence intervals. Hot spots are locations with statistically significant positive z-score clusters at higher than the expected rate, while cold spots are locations with statistically significant negative z-score clusters at lower than the expected rate. Spatial analysis was performed for the health and behavioral categories, age groupings, and methods of disposition to determine whether there were statistically significant hot spots and/or cold spots based on the dogs’ intake locations.

### Statistical analysis

A chi-square test of independence was performed to examine the relation between age group and impound method, as well as the relation between size and disposition. Relationships between environmental conditions (i.e., overall vulnerability by census tracts SVI ranking) and categorical variables (i.e., sex, age group, health category, size group, and dogs that were surrendered to ACCAC by their owners) were assessed through a one-way ANOVA using SAS software (SAS Institute Inc., Version 9.4). Relationships are statistically significant if the p-value < 0.05 for all appropriate methods in this study. Tukey test performed post-hoc analysis of statistically significant categories from the one-way ANOVA results. Results of the Tukey test indicate which characteristic groups were significantly different from other characteristic groups for the same variable.

## Results

ACCAC impounded a total of 3,466 dogs during 2014–2016, representing all age groups (Table 2). The majority of impounded dogs were strays (n = 2,352; 67.9%) followed by dogs whose owners surrendered ownership to ACCAC either in the field or at the shelter (n = 1,018; 29.4%). The remaining dogs were caught by a live trap (n = 61; 1.8%) or their method of arrival was not documented (i.e., other) (n = 35; 1.0%). A chi-square test of independence was performed to examine the relation between age group and impound method. The relation between these variables was significant, *X*^*2*^ (4, N = 3,112) = 13.51, p = 0.009, indicating strong evidence for association.

**Table 2:** Athens-Clarke County Animal Control dog method of arrival, by age group, 2014–2016.

	Method of Arrival				Total
Age Group	Stray^a^	Surrendered^b^	Live Trap	Other ^c^	
**Juvenile**	1249 (66.5%)	576 (30.7%)	37 (2.0%)	17 (0.9%)	**1879 (54.2%)**
**Adult**	916 (70.2%)	352 (27.0%)	24 (1.8%)	13 (1.0%)	**1305 (37.7%)**
**Senior**	170 (64.4%)	89 (33.7%)	0 (0%)	5 (1.9%)	**264 (7.6%)**
**Not available**	17 (94.4%)	1 (5.5%)	0 (0%)	0 (0%)	**18 (0.5%)**
**Total:**	2,352 (67.9%)	1,018 (29.4%)	61 (1.8%)	35 (1.0%)	**3,466**

^a^Stray includes dogs picked up in the field with no owner present or where an owner does not surrender his rights at the time of pick-up and dogs brought to Athens-Clarke County Animal Control by a member of the public but not surrendered.

^b^Surrendered includes dogs in which a human’s ownership claim was surrendered at Athens-Clarke County Animal Control or in the field to an Animal Control Officer.

^c^Other includes instances in which method of impound was not captured.

The final disposition of dogs by size (measured at the time of impound) for all years is included in Table 3. The most common final disposition of dogs was transfer to a rescue group (n = 1,235, 35.6%) followed by adoption (n = 1,026; 29.6%). Twenty-three percent of dogs were reclaimed by their owners (n = 795). For all years, 11% of impounded dogs were euthanized for space, behavioral, or health reasons and 0.8% (n = 28) were disposed of in other ways (arrived at the shelter dead, died of natural causes while in shelter custody, escaped, or stolen). Except where medically recommended, euthanasia of an animal often occurs for a myriad of reasons; a dog with moderate or controllable behavioral issues may not be euthanized following an evaluation if space and other resources for its care are available so conclusions about reason for euthanasia cannot always be drawn from the available data.

**Table 3:** Athens-Clarke County Animal Control, final disposition of impounded dogs by size group at time of intake, 2014–2016.

	Final disposition					Total
Size groups	Transferred to rescue group	Adopted	Reclaimed by owner	Euthanized	Other ^a^	
**Extra small**	210 (57.0%)	99 (27.0%)	44 (11.9%)	13 (3.5%	2 (0.5%)	**368 (10.6%)**
**Small**	467 (45.8%)	325 (32.0%)	157 (15.4%)	60 (6.0%)	10 (1.0%)	**1019 (29.4%)**
**Medium**	312 (33.0%)	325 (34.3%)	181 (19.1%)	125 (13.2%)	4 (0.4%)	**947 (27.3%)**
**Large**	168 (19.4%)	227 (26.3%)	315 (36.5%)	146 (17.0%)	8 (0.9%)	**864 (25.0%)**
**Extra large**	0 (0.0%)	4 (19.0%)	14 (66.7%)	3 (14.3%)	0 (0.0%)	**21 (0.6%)**
**Not available**	78 (31.6%)	46 (18.6%)	84 (34.0%)	35 (14.2%)	4 (1.6%)	**247 (7.1%)**
**Total:**	1,235 (35.6%)	1,026 (29.6%)	795 (23.0%)	382 (11.0%)	28 (0.8%)	**3,466**

^a^Other includes instances where a dog arrived at the shelter dead, died while at the shelter other than by euthanasia, and dogs that escaped or were stolen.

At the time of intake, the majority of dogs were categorized as healthy (n = 2,284, 54.1%); the next most common category was dogs with known health issues (n = 993, 23.5%) (Table 4). Dogs with visible health issues typically associated with physical neglect represented 15.0% (n = 629) of cases. Behaviors associated with social neglect were noted in 6.4% (n = 269) of the dogs impounded. Visible health issues typically associated with trauma (often attributed by staff to vehicular accidents) were present in 1.1% (n = 46) of impounds. Incomplete or inconsistent data collection concerning behavioral evaluations may have led to an underreporting of socially neglected animals. Moreover, evaluations of physical health are prioritized over behavioral evaluations at the time of impound as an animal in pain or fearful does not behave as it normally would. Negative physical conditions can receive immediate veterinary care to resolve the issue, whereas negative behavioral conditions are generally only resolved in the shelter setting through time or euthanasia.

**Table 4:** Athens-Clarke County Animal Control, health category of impounded dogs at time of intake, by year, 2014–2016.

Health or behavioral category at time of intake^a^	Year			Total
	2014	2015	2016	
Healthy	804	814	666	**2,284 (54.1%)**
Known health issue upon intake	355	333	305	**993 (23.5%)**
Visible health issues typically associated with physical neglect	228	212	189	**629 (15%)**
Behaviors typically associated with social neglect	97	103	69	**269 (6.4%)**
Visible health issues typically associated with vehicular trauma	14	21	11	**46 (1.1%)**
**Total:**	1,498	1,483	1,240	**4,221**

^a^Details of health categories are available in Table 1. Dogs may be categorized in more than one health or behavioral category.

**Fig 1:**
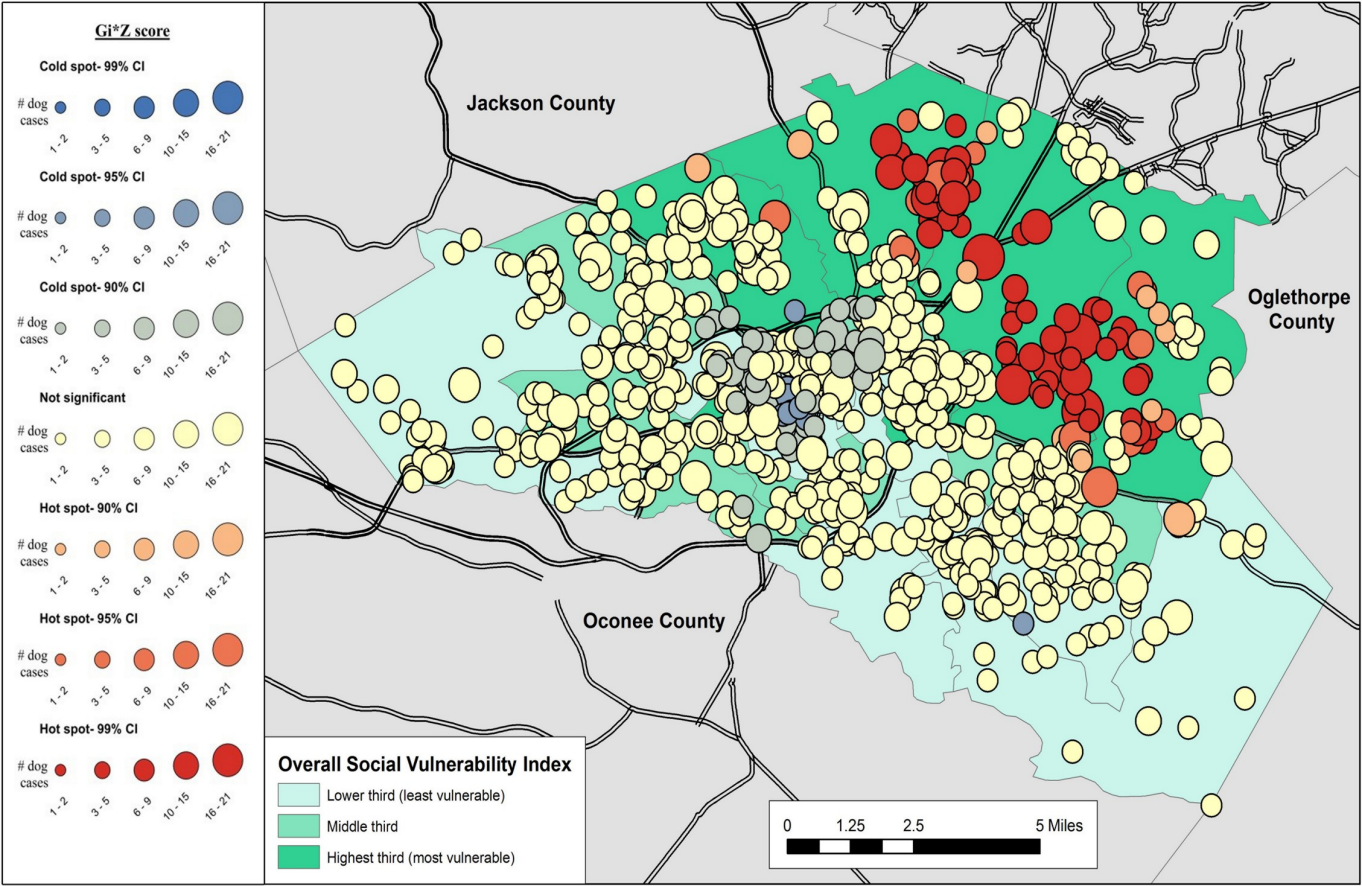
Spatial cluster analysis of dog intake data from Athens-Clarke County Animal Control, Athens-Clarke County, GA, by overall social vulnerability index (SVI), 2014–2016.

**Fig 2:**
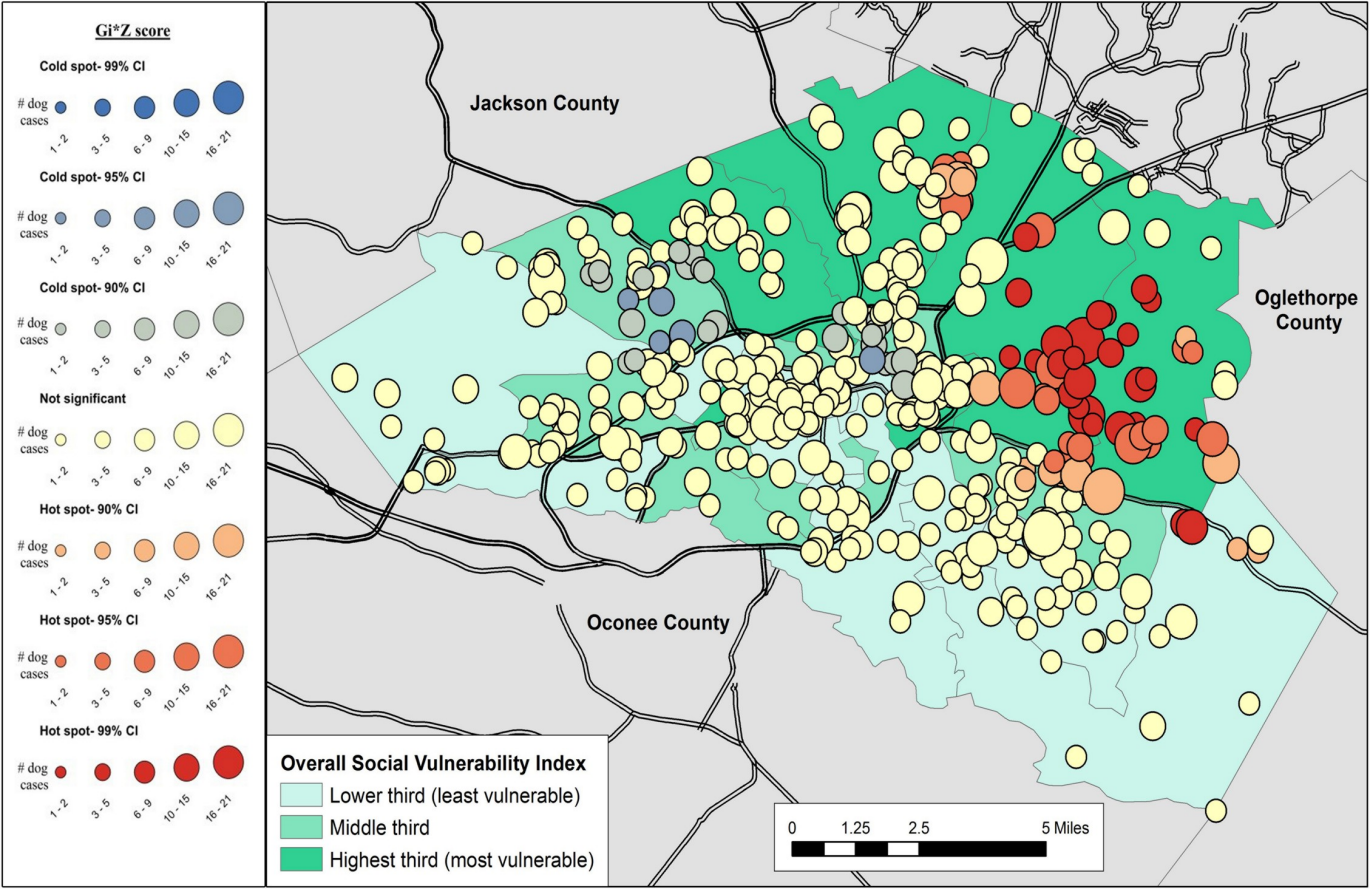
Spatial cluster analysis within the health or behavioral category of known health issues dog intake data from Athens-Clarke County Animal Control, Athens-Clarke County, GA, by overall social vulnerability index (SVI), 2014–2016.

**Fig 3:**
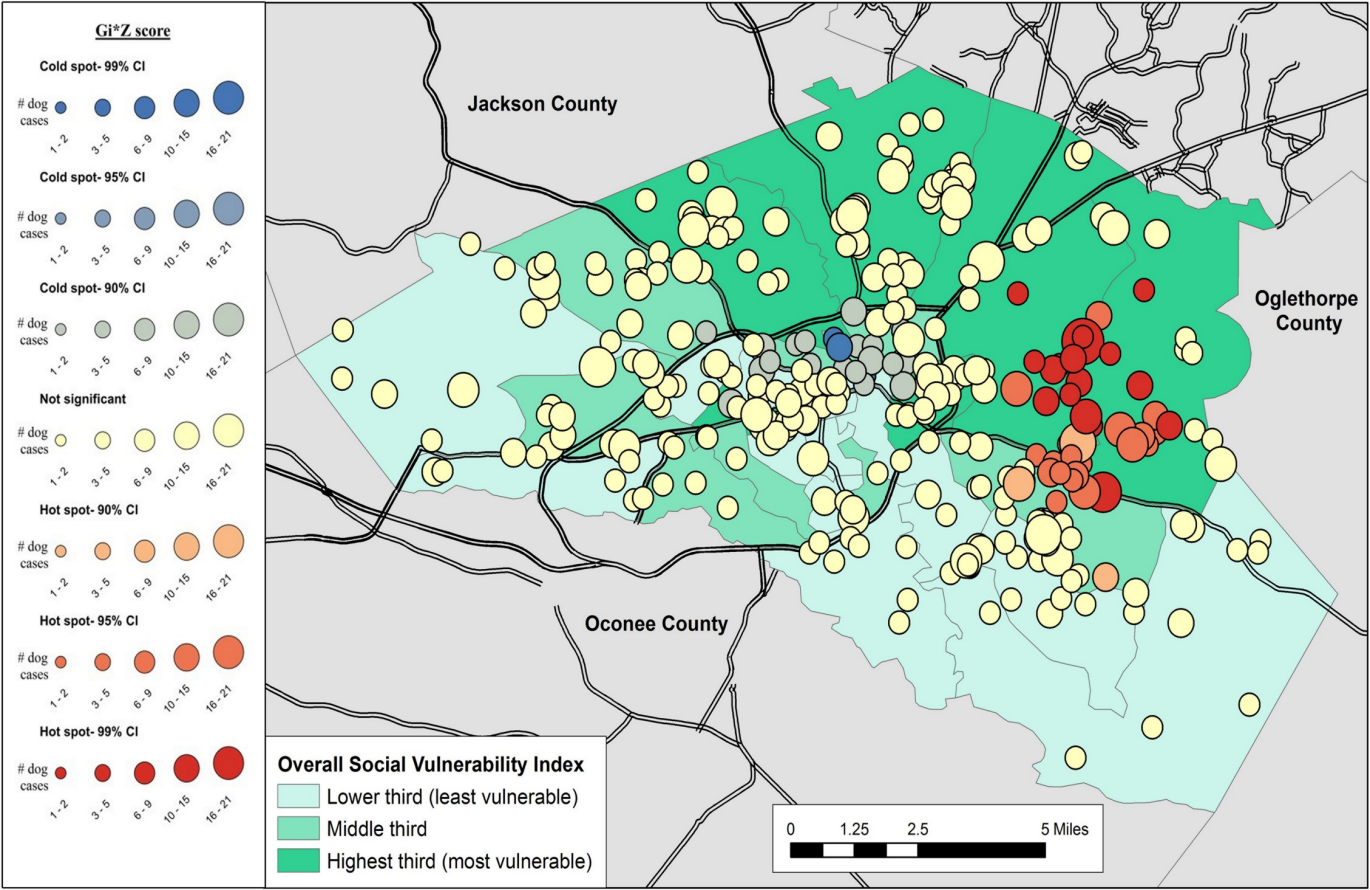
Spatial cluster analysis within the health or behavioral category of physically neglected dog intake data from Athens-Clarke County Animal Control, Athens-Clarke County, GA, by overall social vulnerability index (SVI), 2014–2016.

**Fig 4:**
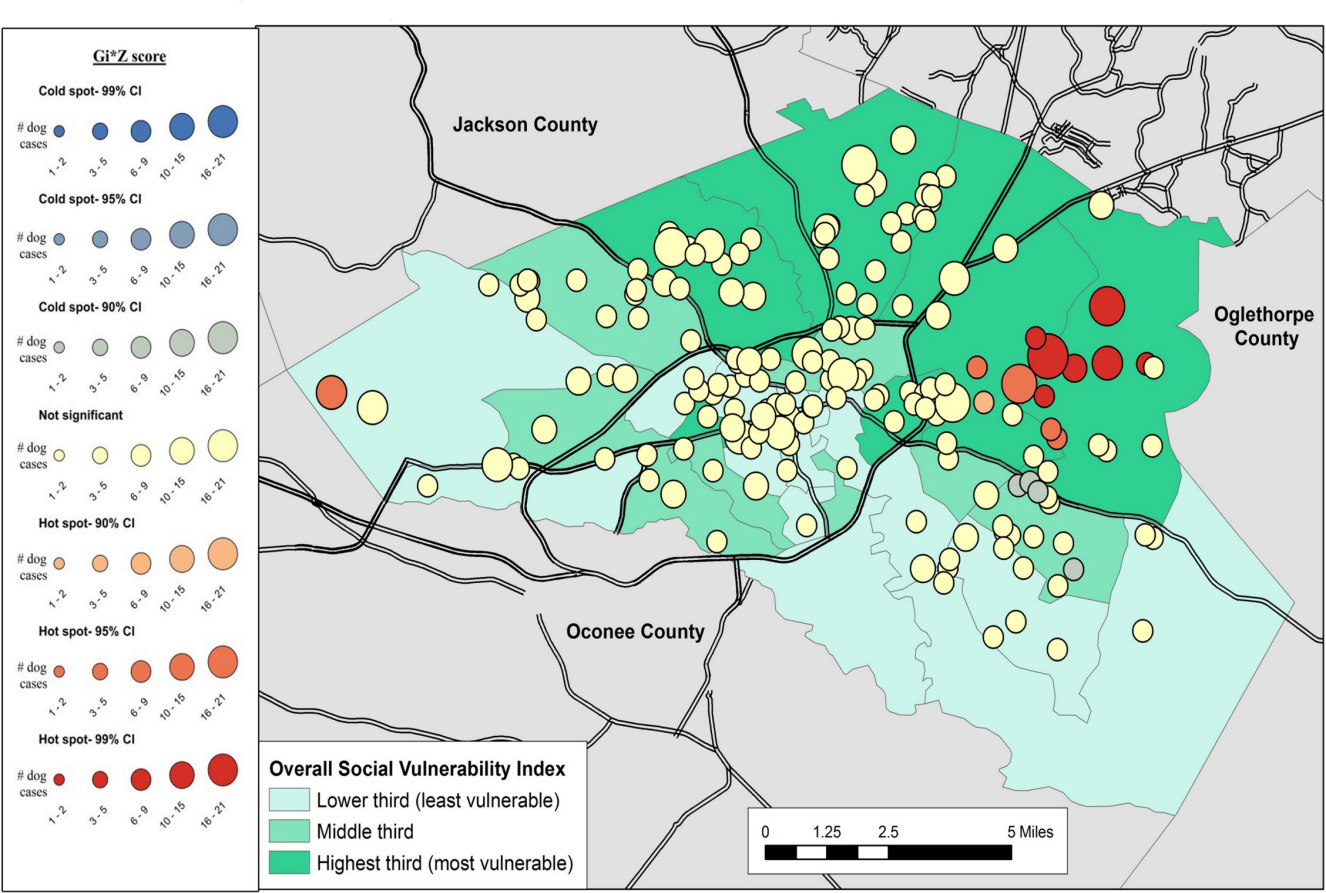
Spatial cluster analysis within the health or behavioral category of socially neglected dog intake data from Athens-Clarke County Animal Control, Athens-Clarke County, GA, by overall social vulnerability index (SVI), 2014–2016.

**Fig 5:**
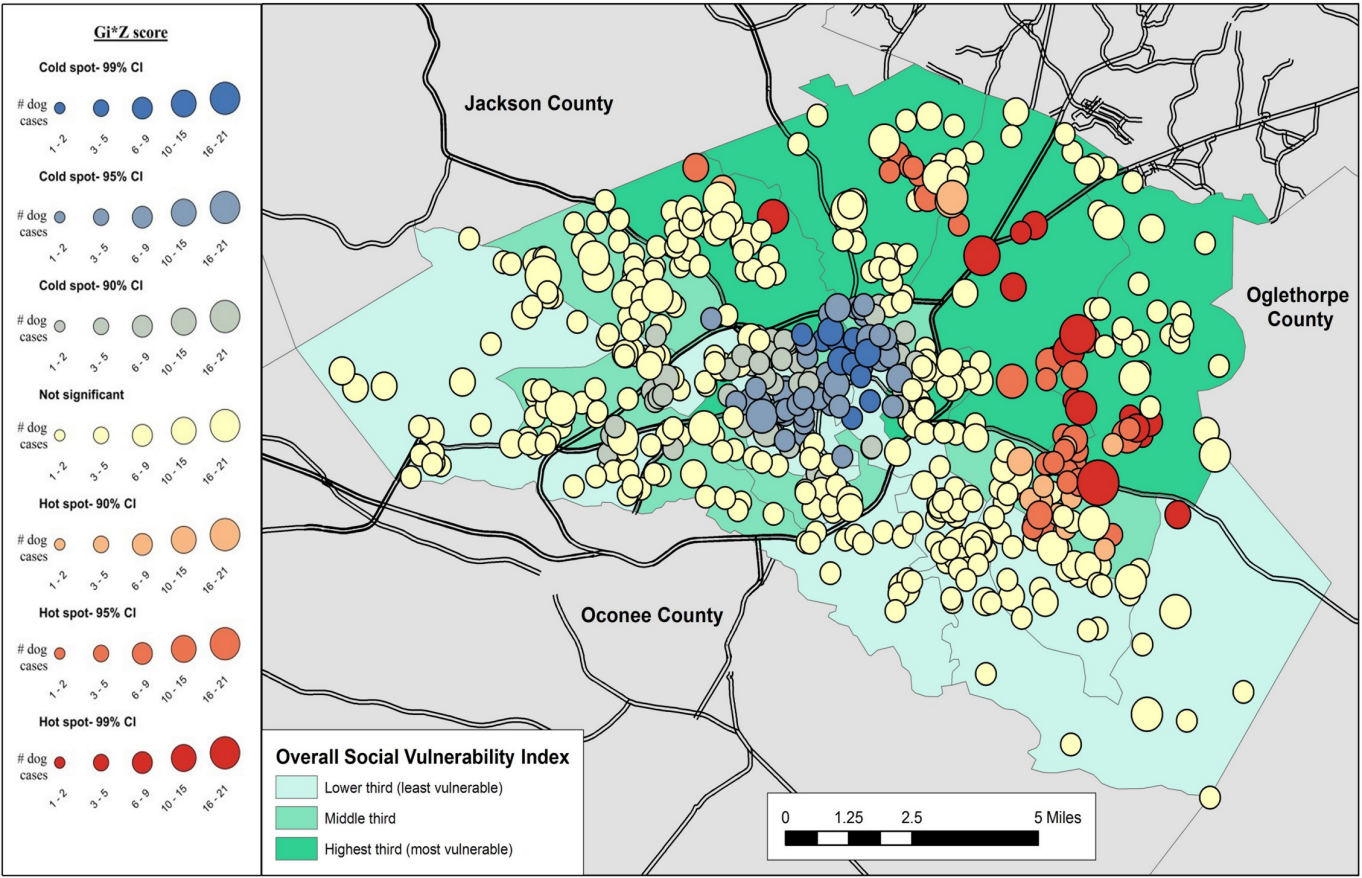
Spatial cluster analysis of adult dog intake data from Athens-Clarke County Animal Control, Athens-Clarke County, GA, by overall social vulnerability index (SVI), 2014–2016.

**Fig 6:**
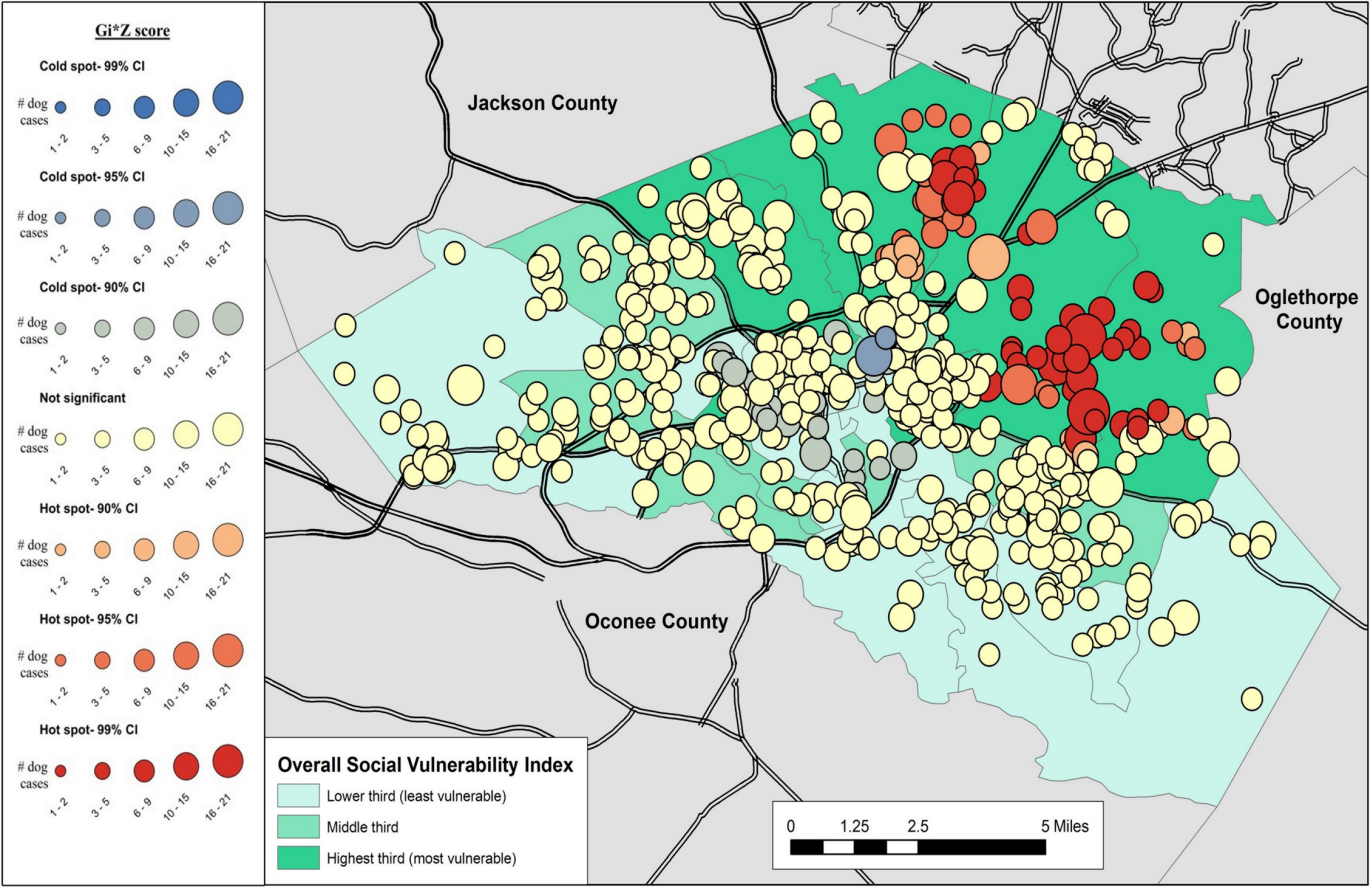
Spatial cluster analysis of juvenile dog intake data from Athens-Clarke County Animal Control, Athens-Clarke County, GA, by overall social vulnerability index (SVI), 2014–2016.

**Fig 7:**
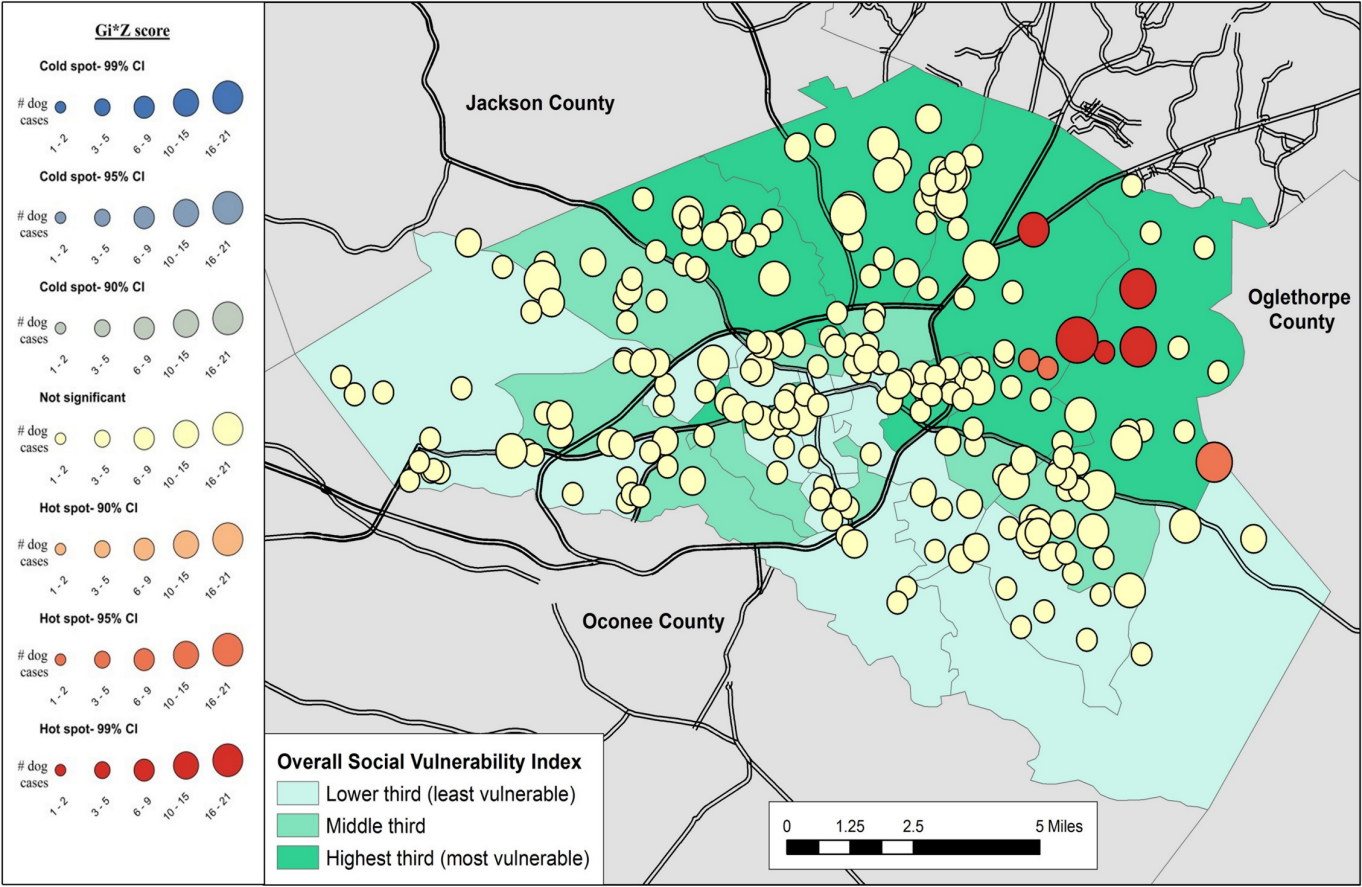
Spatial cluster analysis of dogs euthanized due to severe health issues or behavioral issues within dog intake data from Athens-Clarke County Animal Control, Athens-Clarke County, GA, by overall social vulnerability index (SVI), 2014–2016.

Results of the cluster analysis are in Figs 1–7. Overall distribution of intake clusters is shown in Fig 1, with statistically significant hot spots found to the north and east of Athens. The distribution of clusters produced from the intake locations of dogs in the senior age group and the health categories of healthy and health issues associated with trauma were not statistically significant. Dogs in the health category of known health issues formed statistically significant hot spots to the east of Athens (Fig 2), while those in the health category of physically neglected formed both statistically significant hot spots and cold spots, primarily on the east side of Athens-Clarke County, and primarily located over central Athens, respectively (Fig 3). Dogs categorized as socially neglected formed statistically significant hot spots, located on the east side of Athens-Clarke County and on the south edge of downtown Athens (Fig 4). The spatial distribution of juvenile and adult dog intakes also formed statistically significant hot spots, primarily to the east and north of Athens-Clarke County, and cold spots, primarily located over central Athens (Figs 5 and 6). Compared to other characteristics, age group characteristics formed a greater number of statistically significant clusters throughout Athens-Clarke County. Dogs whose final disposition was euthanasia due to severe health issues or behavioral issues formed only statistically significant hot spots, which were located on the east side of Athens-Clarke County (Fig 7).

Overall mean SVI for Athens-Clarke County U.S. Census tracts ranged from 0.1789–0.9969 (Std. dev. 0.2723; mean 0.4480) percentile rank at the state level. Results of the one-way ANOVA found statistically significant differences between the mean overall SVI of dog intake location and method of intake (p value = 0.0044), age group (p value <0.001), size group (p value <0.001), health or behavioral category (p value = 0.0296), final disposition (p value <0.000), and reason for euthanasia (p value = 0.018) (Table 5).

**Table 5:** ANOVA analysis of Athens-Clarke County Animal Control dog intake characteristics for overall Social Vulnerability Index ranking, 2014–2016.

		Overall SVI^a^			
Characteristic		N	Mean	SD	One-way ANOVA results
**Method of intake**					F = 5.65 p = 0.0044**
	Owner surrender	935	0.5844	0.2866	
	Stray	2,141	0.5821	0.2794	
	Live trap	53	0.7053	0.2626	
**Age group**					F = 15, p <0.001**
	Juvenile	1,711	0.6089	0.2810	
	Adult	1,191	0.5524	0.2780	
	Senior	241	0.5643	0.2872	
**Size group**					F = 8.18, p <0.001**
	Extra small	337	0.6131	0.2880	
	Small	941	0.6060	0.2853	
	Medium	851	0.5713	0.2790	
	Large	800	0.5471	0.2750	
Health or behavioral category***					F = 2.53, p = 0.0296**
	Behaviors typically associated with social neglect	206	0.6328	0.2739	
	Visible health issues typically associated with vehicular trauma	44	0.6100	0.2819	
	Visible health issues typically associated with physical neglect	522	0.5838	0.2865	
	Visible health issues typically associated with physical neglect and behaviors typically associated with social neglect	51	0.6216	0.3016	
	Known health issue upon intake	391	0.6062	0.2836	
	Healthy	1,946	0.5735	0.2795	
**Final disposition**					F = 5.63, p <0.000**
	Adopted	846	0.5828	0.2840	
	Euthanized	381	0.6115	0.2826	
	Transferred to animal rescue group	724	0.5508	0.2677	
	Reclaimed by owner	1,181	0.5959	0.2873	
**Reason for euthanasia**					F = 3.29, p = 0.018**
	Fought with another dog on property	16	0.5754	0.2797	
	Medical necessity	131	0.6671	0.2784	
	Reactivity	99	0.6348	0.2743	
	Space	12	0.5274	0.3248	
	Unknown	123	0.5464	0.2787	

^**a**^Overall Social Vulnerability Index (SVI) obtained from https://svi.cdc.gov. Overall mean SVI for Athens-Clarke County U.S. Census tracts ranged from 0.1789–0.9969 (Std Dev 0.2723; mean 0.4480) percentile rank at the Georgia state ranking. A higher mean SVI represents greater vulnerability, with the most vulnerable equaling one and the least vulnerable equaling zero.

Post hoc comparisons showed several statistically significant differences between the means of the overall SVI for some of both the intake and final disposition categories (Table 6). For intake categories, results of post hoc comparisons indicated statistically significant differences between overall mean SVI of intake location of socially neglected dogs when compared with dogs with known health issues upon intake; extra small dogs compared to large dogs; small dogs compared to medium dogs; small dogs compared to large dogs; physically neglected dogs compared to dogs that were healthy or whose health status was unknown; and juvenile dogs as compared with adult dogs. For disposition categories, the mean overall SVI had a statistically significant difference for dogs euthanized due to medical necessity compared to dogs that were euthanized for unknown reasons; dogs euthanized for behaviors typically associated with social neglect compared to dogs reclaimed by their owners; and dogs transferred to a licensed rescue group compared to dogs reclaimed by their owner.

**Table 6:** Post-hoc analysis of one-way ANOVA on Athens-Clarke County Animal Control dog intake characteristics by Tukey test, 2014–2016.

		Overall SVI*		
Characteristics		Mean difference	95% Confidence Interval	
Method of intake- Live trap	Method of intake- Ownership surrendered	0.121**	0.028	0.214
	Method of intake- Stray	0.123**	0.031	0.215
Age group- Juvenile	Age group- Adult	0.057**	0.032	0.081
Size group- Extra small dogs (weight up to 11.9 lbs.)	Size group- Large dogs (weight between 50.0 and 99.9 lbs.)	0.067**	0.012	0.113
Size group- Small dogs (weight between 12.0 to 24.9 lbs.)	Size group- Medium dogs (weight between 25.0 to 49.9 lbs.)	0.035**	0.001	0.069
	Size group- Large dogs (weight between 50.0 and 99.9 lbs.)	0.059**	0.024	0.094
Health or behavioral category- Behaviors typically associated with social neglect	Health or behavioral category- Known health issue upon intake	0.049**	-0.017	0.115
	Health or behavioral category- Healthy or whose health status was unknown	0.059**	0	0.118
Health or behavioral category- Visible health issues typically associated with physical neglect	Health or behavioral category- Healthy or whose health status was unknown	0.048**	-0.066	0.162
Final disposition- Euthanized due to medical necessity	Final disposition- Euthanized for unknown reasons	0.121**	0.025	0.217
Final disposition- Euthanized due to reactivity	Final disposition- Reclaimed by owner	0.061**	0.015	0.106
Final disposition- Transferred to animal rescue group	Final disposition- Reclaimed by owner	0.045**	0.011	0.079

*Overall Social Vulnerability Index (SVI) obtained from https://svi.cdc.gov.

**Statistically significant p-value < 0.05.

## Discussion

Households located in higher overall SVI tracts may have difficulty caring for their dogs in ways that socialize them appropriately. Undersocialization is not uncommon in situations where dogs are tethered or kenneled alone for extended periods of time. These canine living situations may be a product of greater household vulnerability as, for example, renters have less discretion in how dogs are housed than homeowners, or may reflect differing cultural expectations concerning the care of dogs. Moreover, families with fewer socioeconomic resources may be unable to afford fencing or take trips to dog parks, both of which would allow their dogs more normal social interactions with people and other dogs. Due to the social effect on dogs of tethering and the potential for physical danger, Athens-Clarke County ordinances prohibit the unattended tethering of dogs. Thus, some of the same factors that may lead to the social neglect of dogs may increase the rate at which these dogs are either taken by animal control officers for lack of compliance with local ordinances or are surrendered to the shelter to avoid future legal issues. Once impounded, social neglect may manifest as reactivity in the high-stress shelter setting.

Dogs impounded from lower SVI tracts may have received basic or more specialized veterinary care before coming to the shelter than the dogs presenting with behaviors typically associated with social neglect. Greater financial resources, better access to transportation, and fewer language or cultural barriers in interactions with veterinarians all mean these dogs are more likely to appear healthy or have previously-diagnosed health issues at the time of impound. It is not surprising, then, that dogs ultimately euthanized for behaviors typically associated with social neglect were also more likely to have had intake locations within census tracts with higher overall SVI when compared to dogs categorized as these health categories at intake.

Higher rates of juvenile impounds as compared with adult dog impounds in higher SVI tracts are consistent with outdoor housing for dogs being more common in these areas than in lower SVI areas. Lower SVI tracts may be owner-occupied to a greater extent, have rental rates that incorporate the risk of damage from indoor dogs and thus allow dogs to be housed inside more frequently, and might have neighbors more inclined to report dogs for nuisance activities like barking than households in higher SVI tracts. These dogs might also be intact at higher rates than in lower overall SVI tracts because of cultural norms, financial constraints (including work schedule inflexibility), or limited transportation options. The combination of outdoor housing and high rates of intact dogs lead to the production of more juvenile dogs in these areas. Moreover, this higher rate could also represent a higher replacement rate of dogs within these communities; that is, as dogs are impounded, injured, or fall ill they are replaced with new dogs rather than being reclaimed or returned to health because of the array of factors that lead to the greater social vulnerability of these communities. Thus the age trend for dogs in these areas would point downwards as older dogs are replaced with younger ones and puppies produced locally are retained until they begin to mature.

Families that reside in areas of lower overall SVI may be in a better position to reclaim a lost dog, and dogs from these areas may present better at the shelter because of prior physical care or socialization or have different size, age, or other characteristics and thus be more attractive to adopters. Thus, the animals that remain for limited admission shelters and animal rescue groups to choose between to complement the range of dogs surrendered to them are more apt to come from areas with higher overall SVI.

The observation-based behavioral evaluations referenced in this study are subjective and of uncertain or unknown reliability and validity [37]. While ACCAC staff makes efforts not to include characterizations observed by only one person and explores reported issues before recording them, shelter overcrowding may lead to decisions to euthanize based on incomplete behavioral information or records reflecting this information may not be updated after euthanasia as no longer important. Thus, the margin of error in behavioral records can be expected to be greater than within other data recorded.

Final disposition of the overall dog population within ACCAC may not be representative of other open admission animal shelters even within its region. Although ACCAC remains steadily at capacity for dogs throughout the year, the euthanasia of dogs that are healthy and without behavioral issues is rare. However, final dispositions of dog intake cases categorized as physically or socially neglected may be generalizable from ACCAC to other animal control facilities as the constraints on housing, properly caring for, and placing these animals are common to open admission shelters. Health and behavioral categories should be determined from dog intake data from other similar facilities to determine whether this is the case.

Local officials from ACCAC provided the data utilized in this study. Data are limited and may display collection bias as the public primarily requests animal control officers to the scene. While ACCAC is a progressive facility with dedicated staff and high community engagement, community approaches to reporting animal welfare violations vary. Negative public perception of government officials, a lack of neighborhood solidarity, or a sense that issues should be resolved within the community may dissuade individuals from reporting stray animals, animal abuse, or animal neglect to ACCAC. Alternatively, hypervigilance and overreporting, by formal or self-appointed property managers, may occur in some communities and account for reporting variability between superficially similar communities (e.g., those with similar SVIs and animal density). As a result, the dog impound data may be unevenly distributed.

Duplicating this study in other communities may prove more difficult or prohibitively expensive. The availability of similar data varies across counties and states and, even where available, may require a Freedom of Information Act request increasing the time and cost of performing similar research.

## Conclusions

The purpose of this study was to perform a retrospective spatial analysis of dog intake data collected between 2014–2016 to discern whether there were relationships between characteristics (i.e., health variations) and overall SVI. Findings of this study demonstrate non-random distribution of dogs with specific characteristics within Athens-Clarke County. Results of this project indicate the utility of dog intake information to conduct spatial analysis of the distribution of dog characteristics.

Results of this project indicate the utility of collecting and analyzing dog intake information for use in spatial analysis of the distribution of characteristics of impounded dogs. Athens-Clarke County encompasses a wide range of SVI rankings at the Georgia state ranking SVI scores with census tract means as low 0.1789–0.9969 (Std. dev. 0.2723; mean 0.4480). As possible SVI scores range from 0–1, Athens-Clarke County encompasses both the most vulnerable and some of the least vulnerable areas in the state. The majority of statistically significant hot spots of dog characteristics of interest are located in census tracts falling into the upper third of the overall SVI index results, supporting the argument that humans and dogs that share physical and social environments are similarly vulnerable populations.

The factors considered in this study include some that are likely to be affected by targeted neuter campaigns aimed at reducing pet overpopulation (e.g., the intake location of juvenile and sexually intact reproductive-age dogs) as well as those present regardless of overpopulation (negative physical health conditions and social neglect). Athens-Clarke County can use the results of this study to design outreach programs to provide for the specific need in each part of the county. Shelter intake interventions based on such analysis will vary by community intake demographics. Interventions to address social neglect can target puppy and adult socialization, training, bite inhibition, and desensitization while those to address physical neglect and overproduction of juveniles can focus on the provision of low-cost veterinary care, assistance with installing appropriate outdoor confinement that allows for social enrichment, and sterilization resources, as well as, in both cases, education about the social and physical needs of dogs.
